# Quantum Computing in Medicine

**DOI:** 10.3390/medsci12040067

**Published:** 2024-11-17

**Authors:** James C. L. Chow

**Affiliations:** 1 Radiation Medicine Program, Princess Margaret Cancer Centre, University Health Network, Toronto, ON M5G 1X6, Canada; james.chow@uhn.ca; Tel.: +1-416-946-4501; 2 Department of Radiation Oncology, University of Toronto, Toronto, ON M5T 1P5, Canada

**Keywords:** quantum computing, medicine, healthcare, quantum algorithms, quantum machine learning, drug discovery, personalized medicine, medical diagnostics, Monte Carlo simulation, radiotherapy optimization

## Abstract

Quantum computing (QC) represents a paradigm shift in computational power, offering unique capabilities for addressing complex problems that are infeasible for classical computers. This review paper provides a detailed account of the current state of QC, with a particular focus on its applications within medicine. It explores fundamental concepts such as qubits, superposition, and entanglement, as well as the evolution of QC from theoretical foundations to practical advancements. The paper covers significant milestones where QC has intersected with medical research, including breakthroughs in drug discovery, molecular modeling, genomics, and medical diagnostics. Additionally, key quantum techniques such as quantum algorithms, quantum machine learning (QML), and quantum-enhanced imaging are explained, highlighting their relevance in healthcare. The paper also addresses challenges in the field, including hardware limitations, scalability, and integration within clinical environments. Looking forward, the paper discusses the potential for quantum–classical hybrid systems and emerging innovations in quantum hardware, suggesting how these advancements may accelerate the adoption of QC in medical research and clinical practice. By synthesizing reliable knowledge and presenting it through a comprehensive lens, this paper serves as a valuable reference for researchers interested in the transformative potential of QC in medicine.

## 1. Introduction

Quantum computing (QC) is a revolutionary field of computation that leverages the principles of quantum mechanics to process information in ways fundamentally different from classical computers. In QC, qubits are the basic units of quantum information, analogous to the bits used in classical computing [1]. Unlike classical bits, which can be either 0 or 1, qubits take advantage of quantum properties like superposition and entanglement. Superposition allows qubits to exist in a state that represents both 0 and 1 simultaneously, rather than one definite state. This capability enables quantum computers to process a vast amount of information in parallel, potentially solving complex problems faster than classical computers. In addition, qubits can become entangled, meaning the state of one qubit can depend on the state of another, even when separated by large distances [2]. This interconnectedness is unique to qubits and enables complex computational processes that are infeasible with traditional bits. These properties give quantum computers their unique ability to solve certain types of problems—such as factoring large numbers or simulating quantum systems—more efficiently than classical computers.

In high-performance computing, supercomputers and quantum computers serve distinct purposes, employing different underlying principles. Supercomputers use classical computing architectures, relying on vast numbers of processors and advanced algorithms to perform complex calculations at extremely high speeds. In contrast, quantum computers use qubits that can exist in multiple states simultaneously, thanks to superposition and entanglement. This enables quantum computers to process information in ways that are fundamentally different from classical systems, allowing them to tackle specific problems, such as optimization and simulation of quantum systems, exponentially faster than supercomputers. While supercomputers remain powerful tools for a variety of applications, the unique capabilities of QC open new avenues for solving complex challenges, particularly in fields like medicine.

The relevance of QC to medicine lies in its potential to address computational challenges that are currently insurmountable for classical computers. The ability to process and analyze vast datasets, model complex biological systems, and optimize decision-making processes positions QC as a transformative tool in medical research and clinical applications [3]. In areas such as drug discovery, genomics, personalized medicine, Monte Carlo dose calculation, and radiotherapy optimization, QC could significantly accelerate breakthroughs by providing solutions to problems that require massive computational power [4,5,6,7,8]. By harnessing quantum principles, researchers aim to develop more precise and efficient diagnostic tools, enhance treatment plans, and unlock deeper insights into disease mechanisms, ultimately advancing the field of medicine.

The potential of QC to revolutionize healthcare lies in its unparalleled ability to solve complex problems that are beyond the reach of classical computers. One of the most significant challenges in modern medicine is the sheer volume and complexity of data generated by medical research, clinical trials, and patient records [9]. QC can process vast amounts of information simultaneously, making it an ideal tool for data-heavy tasks such as genomics, personalized medicine, and predictive analytics. For example, QC can accelerate the discovery of new drugs by simulating molecular interactions at a scale that classical systems cannot handle efficiently [10]. In addition, quantum algorithms can optimize clinical decision-making processes, leading to more precise diagnoses and treatment recommendations, especially in fields like oncology [11] and cardiology [12], where individual patient data must be processed in real-time to deliver personalized care. Table 1 illustrates the comparative advantages of QC over classical computing in the medical field [2,3,4]. QC offers significantly faster data processing speeds through parallel computations, enabling efficient solutions to complex problems like molecular interactions and genomic analysis. It accelerates drug discovery by enhancing molecular simulations and improves medical imaging through advanced techniques that capture finer details. Moreover, QC optimizes personalized treatment plans by analyzing multiple factors simultaneously while also enhancing AI models for better diagnostics. With the potential to reduce resource requirements and improve data security through advanced encryption, quantum computing represents a transformative force in healthcare, promising faster, more efficient, and more secure medical solutions compared to traditional computing methods.

However, the limitations of classical computing in healthcare are becoming more evident as the complexity of medical challenges grows. Traditional computers struggle with tasks that involve simulating large, intricate systems, such as modeling the interactions between thousands of molecules in drug design or mapping the genetic variations in a patient’s genome for personalized medicine. These problems are typically NP-hard, meaning they cannot be solved in a reasonable timeframe by classical algorithms due to the exponential increase in computational requirements [13]. Moreover, optimizing radiotherapy plans and running highly detailed simulations of human tissues also push the limits of classical computers, resulting in long processing times and less precise outcomes [14,15]. QC promises to overcome these bottlenecks, offering solutions that are not only faster but also more accurate, laying the groundwork for future advances in medical research and clinical practice.

This paper will explore the transformative potential of QC in the field of medicine, focusing on its ability to address complex challenges that classical computing struggles to solve. It will delve into the core principles of QC—such as qubits, superposition, and entanglement [16]—and examine how these principles can be applied to revolutionize medical research, diagnostics, and treatment planning. The paper will also highlight current limitations in healthcare computing and showcase how QC could overcome these barriers, offering faster, more precise solutions to data processing, drug discovery, and personalized medicine. Moreover, the paper will discuss the real-world applications, challenges, and future directions of integrating QC into medical practice. Given space limitations, this review provides an overview of general QC concepts and developments in medicine, rather than an in-depth discussion of any specific topic.

## 2. Historical Overview

### 2.1. Evolution of QC

QC began as a theoretical construct in the 1980s, when physicist Richard Feynman and mathematician David Deutsch laid the groundwork for quantum computation. Feynman, in particular, recognized that classical computers were inadequate for simulating quantum systems, a limitation that inspired the concept of a quantum computer [17]. Deutsch later formalized this concept by proposing the first quantum Turing machine, illustrating that quantum computers could solve certain problems faster than classical machines by exploiting the principles of quantum mechanics—specifically superposition and entanglement [18]. However, the early theoretical models lacked practical applications due to technological constraints in manipulating quantum states [19].

The transition of QC from theory to practical advancements occurred in the late 1990s and early 2000s. One of the most significant developments was the creation of Shor’s algorithm by Peter Shor in 1994, which demonstrated that quantum computers could efficiently factor large numbers, a task that is infeasible for classical computers [20]. This marked the first major demonstration of QC’s superiority in certain computational tasks. In parallel, the development of quantum error correction codes addressed one of the most critical challenges in QC: the inherent fragility of qubits. By minimizing the impact of errors in quantum computations, these codes allowed for the construction of more stable and reliable quantum systems, laying the foundation for future practical applications [21].

### 2.2. Key Breakthroughs in Medicine

The application of QC in medicine began to take shape with advances in quantum algorithms and hardware during the 2010s [22]. One breakthrough that enabled QC’s use in the medical field was the development of quantum algorithms for molecular simulation, which are crucial in drug discovery [23]. Classical computers struggle to simulate the quantum nature of complex molecules due to the vast number of variables involved, as they must account for each possible state of every particle independently. This approach quickly becomes computationally prohibitive, especially for complex molecular systems. Quantum computers, however, can use quantum gates and quantum annealing to model these interactions more accurately and efficiently by leveraging quantum properties like superposition and entanglement. Quantum annealing, in particular, is a QC method that focuses on finding optimal or near-optimal solutions to problems with many possible configurations, which is common in molecular simulations and optimization tasks. Unlike gate-based QC, which relies on sequential operations, quantum annealing uses a process where qubits evolve gradually from an initial state to a final state that represents the optimal solution, guided by quantum tunneling and superposition principles. Companies like Google [24] and IBM [25] began to apply their QC prototypes to molecular simulations, which hold the potential to revolutionize pharmacology by speeding up the discovery of new drugs and reducing the cost of clinical trials.

Another milestone in QC’s relevance to medicine was the advent of quantum machine learning (QML) [26]. As healthcare data become more complex and voluminous, QML offers the potential to process and analyze these data at unprecedented speed and precision. Quantum-enhanced algorithms have already been applied to genomic data analysis, helping researchers identify patterns and correlations that are otherwise difficult to detect with classical methods [27]. These breakthroughs are opening new frontiers in personalized medicine, diagnostics, and treatment optimization, positioning QC as a transformative tool in the medical field.

### 2.3. Timeline of QC in Medicine

QC’s intersection with medicine began to materialize in the early 21st century, as advancements in both quantum theory and hardware laid the groundwork for practical applications in healthcare. One of the first major milestones occurred when quantum algorithms were successfully applied to simulate molecular interactions for drug discovery [28]. This was a pivotal moment, as pharmaceutical companies began collaborating with QC firms to explore how quantum simulations could accelerate the development of new drugs, particularly for complex diseases like cancer and Alzheimer’s [29].

In 2019, another significant milestone was achieved when Google announced quantum supremacy, demonstrating that its quantum computer, Sycamore, could perform a specific task faster than any classical supercomputer [30]. While this experiment was not directly related to medicine, it signaled that quantum computers were becoming powerful enough to tackle real-world problems, including those in medical research [31]. Around the same time, the first practical applications of QML emerged, offering powerful tools for medical diagnostics and genomics. Early QML algorithms were tested for their ability to process large datasets in healthcare, demonstrating the potential to outperform classical machine learning models in identifying patterns within genomic data, protein structures, and radiology images [32].

The year 2021 saw a significant leap forward with the use of quantum-enhanced algorithms for optimizing radiotherapy treatment plans. Traditional radiotherapy requires complex calculations to determine the optimal radiation dose for a tumor while minimizing damage to surrounding tissues [11]. QC’s ability to process multiple variables simultaneously enabled more precise and efficient treatment plans, potentially reducing treatment times and improving patient outcomes. This marked the first real integration of QC into clinical practice, demonstrating its potential to directly impact patient care [33].

**Figure 1:**
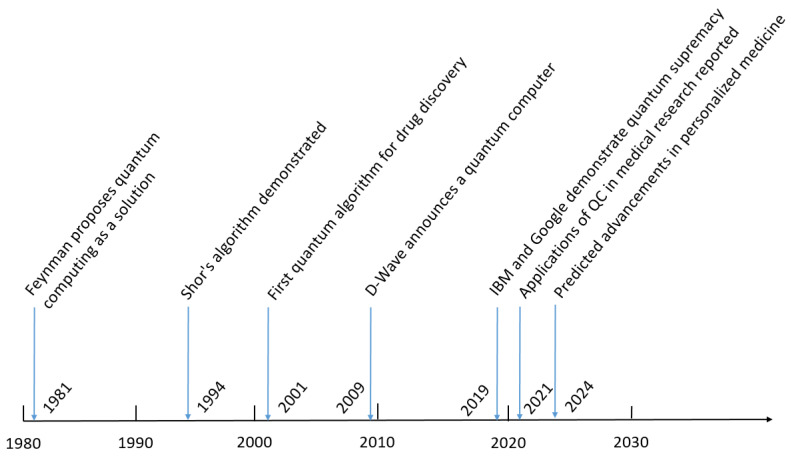
Timeline of key milestones in quantum computing’s evolution in medicine, from Feynman’s 1981 proposal to anticipated advancements in personalized medicine by 2024. Notable events include Shor’s algorithm in 1994, the first drug discovery algorithms in 2001, and IBM and Google’s demonstration of quantum supremacy in 2019, highlighting the transformative potential of quantum computing in healthcare.

Moreover, several ongoing research initiatives are exploring the use of QC in personalized medicine, where quantum algorithms are being tested to predict individual patient responses to drugs based on genetic profiles [34]. This approach could revolutionize how treatments are tailored to patients, offering more accurate and effective therapies. With continued advancements in quantum hardware and algorithms, the next decade promises to see even deeper integration of QC into the medical field, transforming how we approach disease modeling, treatment optimization, and healthcare delivery. Figure 1 summarizes the timeline of QC in medicine.

## 3. QC Techniques in Medical Research

### 3.1. Quantum Algorithms for Drug Discovery

QC has introduced powerful algorithms that have the potential to revolutionize drug discovery by enabling more efficient molecular modeling. One such algorithm is Grover’s algorithm, which is designed to search unsorted databases more quickly than classical algorithms [35]. In the context of drug discovery, this algorithm can be used to scan vast chemical databases to identify potential drug candidates that match specific molecular criteria. Another key quantum algorithm applied in this domain is the variational quantum eigensolver (VQE), which is particularly useful for simulating the electronic structure of molecules [36]. VQE leverages the principles of quantum mechanics to find the lowest energy state, or ground state, of a molecule—a critical task in predicting how molecules will interact in biological systems. Classical computers struggle with this type of simulation as molecular complexity increases, but quantum computers can more accurately model these interactions, significantly reducing the time and cost of the drug development process. These algorithms offer the promise of more precise molecular simulations, which could lead to the discovery of new therapeutic compounds for complex diseases [37].

### 3.2. QML in Healthcare

QML represents a promising frontier in healthcare by enhancing the capabilities of traditional machine learning models [38] through the power of QC. QML leverages quantum algorithms to process and analyze large, complex datasets more efficiently than classical systems. One of the applications is in diagnostic analytics, where quantum-enhanced models can detect patterns in medical data, such as radiological images, with greater speed and precision [39]. For instance, QML can improve the accuracy of early cancer detection by analyzing medical images or genomic data, helping doctors identify subtle indicators of disease that classical models might miss [40]. In addition, QML is making strides in predictive analytics, enabling more accurate predictions of disease progression and patient outcomes [41]. By analyzing large datasets of patient histories, genetic profiles, and treatment responses, QML models can provide deeper insights into individualized healthcare plans, leading to more personalized and effective treatments. These quantum-enhanced models not only increase the speed of computation but also enhance the accuracy of medical decision-making, paving the way for more sophisticated diagnostic tools and predictive systems in healthcare.

### 3.3. Quantum Imaging Techniques

Quantum principles have the potential to revolutionize medical imaging by enhancing the precision and resolution of imaging technologies like magnetic resonance imaging (MRI). Traditional MRI relies on the interaction of magnetic fields and radio waves to create images of the body’s internal structures [42], but QC and quantum sensors show promise in significantly improving the accuracy of these scans. One advancement in this area is the development of quantum-enhanced MRI, which uses quantum coherence and entanglement to generate higher-resolution images, enabling earlier and more accurate detection of abnormalities such as tumors [43]. Quantum sensors, based on quantum entanglement, can detect minute magnetic fields with greater sensitivity than classical sensors, making it possible to capture more detailed images of soft tissues and neural networks [44]. These advancements not only improve the quality of imaging but also reduce the amount of time required for a scan, potentially lowering patient discomfort and exposure to magnetic fields. While quantum imaging techniques are not directly related to QC, they continue to advance and hold significant potential for enhancing diagnostic capabilities, enabling more precise and non-invasive medical evaluations.

### 3.4. Quantum-Optimized Treatment Plans

QC holds great promise in optimizing treatment plans for radiotherapy and personalized medicine by offering unprecedented computational power for complex calculations. In radiotherapy, the goal is to precisely target cancerous tissues with minimal damage to surrounding healthy tissues, a process that involves calculating the optimal dose distribution of radiation [45]. Quantum computers, with their ability to process large, multidimensional datasets simultaneously, can optimize these dose distributions far more efficiently than classical methods. This enables more accurate and personalized radiation treatments, reducing side effects and improving patient outcomes. In the field of personalized medicine, quantum approaches can be used to tailor treatments based on an individual’s genetic profile [46]. Quantum algorithms can analyze vast amounts of genetic and clinical data to predict how a patient will respond to specific therapies, allowing for the development of highly personalized treatment plans. By leveraging the computational advantages of quantum systems, healthcare providers can optimize therapies in real-time, improving both the precision and effectiveness of medical interventions [47].

## 4. Practical Applications of QC in Healthcare

**Figure 2:**
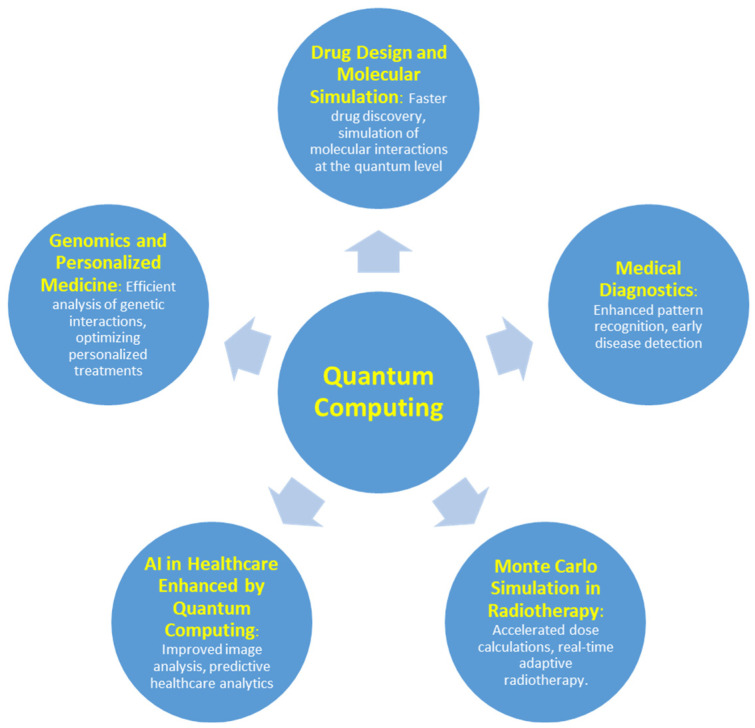
Schematic diagram showing the key applications of QC in medicine, including drug design and molecular simulation, genomics and personalized medicine, medical diagnostics, AI-enhanced healthcare, and Monte Carlo simulations in radiotherapy. QC’s advanced computational capabilities offer transformative potential in improving accuracy, speed, and efficiency across these critical healthcare domains.

Figure 2 shows the diverse applications of QC in medicine, highlighting its potential to revolutionize fields such as drug design, genomics, medical diagnostics, AI-enhanced healthcare, and radiotherapy through enhanced computational power and efficiency. Each branch represents a key area where QC is poised to make significant advancements, improving both precision and speed in medical research and clinical practice.

### 4.1. Drug Design and Molecular Simulation

QC is revolutionizing drug design and molecular simulation by significantly accelerating the drug discovery process. Traditional methods for simulating molecular interactions and predicting how a drug will interact with biological targets are computationally expensive and time-consuming, especially for complex molecules [48]. Quantum computers, however, can simulate these interactions at the quantum level, providing more accurate models of molecular behavior in significantly less time than classical computers [49]. This enables researchers to identify promising drug candidates more quickly and with greater precision, thereby reducing the time and cost associated with drug development. One notable case study involves Biogen’s collaboration with Accenture Labs, where quantum algorithms were applied to expedite the discovery of treatments for neurological diseases like Alzheimer’s, Parkinson’s, and Lou Gehrig’s disease [50]. Moreover, Moderna has partnered with IBM to explore QC for studying mRNA used in vaccines [51]. These real-world examples demonstrate how quantum simulations are not just theoretical tools but are actively transforming the pharmaceutical industry by streamlining the drug development pipeline and potentially bringing life-saving treatments to market faster [52].

### 4.2. Genomics and Personalized Medicine

QC is poised to transform genomics and personalized medicine by enabling the analysis of complex genetic interactions at a scale that is currently impossible with classical computers [53]. The intricate nature of human genetics, with its vast amount of data and interdependent factors, requires immense computational power to understand how genes interact with each other and influence disease. Quantum algorithms can model these complex interactions more efficiently, identifying patterns and genetic mutations that contribute to diseases like cancer, Alzheimer’s, and heart disease [54]. In the realm of personalized medicine, quantum models offer the ability to optimize treatment plans by processing vast datasets, such as a patient’s genomic information, medical history, and environmental factors, to tailor treatments that are specific to the individual’s genetic makeup. This personalized approach improves treatment outcomes by ensuring that therapies are not only targeted but also adaptable to a patient’s unique genetic profile. For example, collaborations like Cambridge QC’s efforts to use quantum technology for genetic research and Strelchuk et al.’s [55] partnership with the Q4Bio program to study mapping DNA diversity using QC.

### 4.3. Medical Diagnostics

QC is emerging as a powerful tool in medical diagnostics, particularly through its ability to enhance pattern recognition and data analysis for the early detection of diseases. Quantum algorithms, such as quantum neural networks [56] and quantum support vector machines [57], have the potential to process complex medical datasets more efficiently than classical systems, identifying subtle patterns that are often missed. This capability is especially useful in diagnosing diseases like cancer and neurodegenerative disorders, where early detection is critical for successful treatment outcomes. For instance, QC can be used to analyze vast amounts of imaging data, such as MRI scans, or genetic data to identify early markers of diseases like Alzheimer’s or Parkinson’s [58]. In oncology, quantum algorithms can assist in detecting cancerous cells at earlier stages by recognizing unique patterns in imaging or genomic data that are too complex for classical computers to process effectively. Google’s Quantum AI team [59] and D-Wave [60] have been actively researching how QC can accelerate early cancer diagnosis through enhanced pattern recognition and the analysis of large medical datasets, paving the way for more accurate and timely diagnostics. In D-Wave’s implementation, quantum speedup refers to the ability of quantum annealers to solve certain types of complex optimization problems faster than classical algorithms or traditional supercomputers. D-Wave’s system leverages quantum tunneling, a unique quantum phenomenon where qubits can transition between states even if an energy barrier exists between them. This enables the system to explore many possible solutions simultaneously, leading to a significant reduction in the time needed to reach the optimal solution compared to classical brute-force methods.

### 4.4. AI in Healthcare Enhanced by QC

The integration of QC with AI has the potential to significantly enhance the capabilities of AI models in healthcare. Quantum AI can process vast and complex datasets more efficiently than classical AI models, leading to improvements in areas like radiology, where quantum-enhanced algorithms can provide more accurate image analysis and diagnostics [61]. QC can also elevate predictive healthcare analytics by enabling AI models to analyze large, multidimensional datasets—such as patient histories, genetic data, and environmental factors—faster and with greater precision. This allows for better predictive modeling, which can identify patients at risk for specific diseases or complications earlier and more accurately [62]. Additionally, quantum-enhanced AI models can be used to optimize treatment plans and provide real-time insights into patient responses to therapies. Companies like IBM, Google, and Rigetti Computing are at the forefront of research that combines QC with AI to drive advancements in healthcare, with applications ranging from medical imaging to personalized medicine and drug discovery [63].

### 4.5. Monte Carlo Simulation in Radiotherapy

The Monte Carlo simulation is a powerful computational technique widely used in radiotherapy for accurate dose calculation and treatment planning. The stochastic nature of the Monte Carlo method allows it to simulate the complex interactions of radiation particles with matter, providing highly precise models of radiation dose distribution within a patient’s body [64]. This is particularly crucial for advanced radiotherapy techniques such as intensity-modulated radiation therapy and proton therapy, where precise dose calculations are necessary to maximize tumor control while minimizing damage to healthy tissues [65,66]. The Monte Carlo simulation accounts for various physical phenomena, including scattering, absorption, and secondary particle production, making it one of the most reliable methods for calculating radiation doses in heterogeneous tissues, such as those found in the lungs or head and neck [67,68,69]. Although Monte Carlo simulations are computationally intensive, advancements in high-performance computing and QC are reducing calculation times, making this technique more accessible for clinical use [70]. By integrating quantum algorithms into Monte Carlo simulations, researchers can further accelerate these calculations [71], potentially enabling real-time adaptive radiotherapy that dynamically adjusts treatment plans based on the patient’s anatomy during treatment.

## 5. Challenges and Current Limitations

Despite its immense potential, QC faces significant technological barriers that limit its current application in medicine and healthcare. One of the primary challenges is the limited hardware capabilities of quantum computers. Current quantum processors, such as those developed by IBM and Google, are still in the noisy intermediate-scale quantum (NISQ) era, where quantum bits, or qubits, are highly susceptible to errors due to decoherence and noise from environmental interactions [72]. This limits the scalability of quantum systems, as even a small number of qubits can become difficult to control and maintain in a stable state [73]. Moreover, the need for highly controlled environments, such as extremely low temperatures and vacuum conditions, makes quantum hardware difficult and expensive to develop. These scalability issues also extend to the number of qubits required to solve real-world medical problems, as large-scale simulations for drug discovery, personalized medicine, or radiotherapy would require thousands to millions of fault-tolerant qubits—far beyond the capabilities of current hardware [74]. Addressing these technological barriers will be critical to unlocking the full potential of QC in healthcare, and ongoing research is focused on improving qubit stability, error correction, and hardware scalability.

Integrating QC into clinical settings poses significant challenges due to the complexity of quantum systems and the existing healthcare infrastructure. Unlike classical computing systems, which are well-established in hospitals and research institutions, quantum computers require specialized environments, such as ultra-low temperatures and isolated conditions, making them difficult to install and maintain within standard clinical facilities [75]. In addition, quantum algorithms are not yet fully compatible with the current healthcare IT systems, which are built around classical computing architectures. This creates hurdles in data integration, as patient records, diagnostic images, and treatment planning systems rely on classical data formats and software. QC also requires a highly skilled workforce capable of understanding both quantum mechanics and clinical applications, making the training of medical professionals a key challenge. Furthermore, issues related to regulatory approval, as well as ensuring the accuracy and reliability of quantum-driven healthcare tools, must be addressed before these systems can be used in clinical decision-making [62]. Until QC systems can be seamlessly integrated with existing medical infrastructure and processes, their implementation in clinical settings will remain a long-term goal rather than a near-term reality [2,13].

As QC advances, its powerful data processing capabilities raise significant ethical and data privacy concerns, particularly in the context of healthcare. Quantum computers could potentially decrypt data at speeds far beyond what is possible with classical computing, posing a threat to the security of sensitive patient data if proper safeguards are not in place [76]. The ability of quantum systems to break current encryption methods (such as RSA and other cryptographic protocols) highlights the need for quantum-safe encryption to protect medical records and personal health information [77]. This creates a dilemma for healthcare institutions that must balance the benefits of QC—such as faster diagnostics and treatment optimization—with the potential risks of data breaches and cybersecurity vulnerabilities. Moreover, ethical concerns arise over data ownership, consent, and the transparency of quantum algorithms in healthcare. For example, quantum-enhanced AI models used for medical decision-making could lead to issues of algorithmic bias and explainability, challenging healthcare providers to ensure that these tools are used fairly and equitably [78,79]. Addressing these ethical and privacy concerns is essential to ensuring that QC can be integrated into healthcare in a way that respects patient rights and upholds the highest standards of data security.

One of the major barriers to the widespread adoption of QC in healthcare is the high cost associated with quantum hardware and its maintenance. Quantum computers require highly specialized and expensive components, such as superconducting qubits or trapped ions, which operate in extreme environments like cryogenic temperatures or ultra-high vacuum conditions. These setups necessitate significant investments in infrastructure, cooling systems, and ongoing operational costs, making QC far more expensive than classical computing [80]. For healthcare institutions, many of which already operate under tight budgets, the cost of installing and maintaining quantum systems is prohibitive, especially given the need for specialized staff to oversee and manage these systems. Additionally, the limited availability of quantum computers means that access is often restricted to a few research institutions or corporations, further driving up costs due to shared usage models. This raises concerns about economic inequality, as only well-funded organizations may be able to afford the benefits of QC, potentially leaving others behind. Until quantum hardware becomes more affordable and scalable, its adoption in healthcare will remain constrained by economic and resource limitations [13]. Table 2 summarizes the current challenges and limitations of implementing QC in medicine. It is seen that while QC holds great promise, significant technological, logistical, and ethical barriers must be overcome. These challenges include limited hardware capabilities, difficulties with integration into clinical settings, high costs, and concerns over data privacy and algorithmic fairness. Addressing these issues will be essential for the widespread adoption of quantum computing in healthcare. To align QC with healthcare IT systems, key steps include establishing interoperability standards to enable QC and classical systems to work together, particularly for integration with electronic health records. Developing a hybrid computing framework will allow QC to handle specific tasks, like complex diagnostics or treatment optimization, while classical systems manage standard operations. Data privacy frameworks tailored for QC, ensuring GDPR and HIPAA compliance [81], are crucial to safeguard patient information. Addressing algorithmic fairness will also help mitigate biases in QC-powered healthcare algorithms. Moreover, training healthcare staff in QC concepts and piloting targeted applications, such as diagnostic imaging analysis, will allow QC integration to proceed in a controlled and impactful way.

## 6. Future Directions

One of the key areas driving the future of QC in healthcare is the development of quantum error correction and other hardware innovations. Error correction is critical because qubits, the fundamental units of quantum computers, are highly sensitive to noise and decoherence, which lead to errors during computations [82]. Recent advancements in fault-tolerant QC aim to address these issues by using quantum error correction codes that detect and fix errors without disrupting the computational process. As these technologies mature, they will significantly enhance the stability and scalability of quantum systems, enabling more complex and reliable quantum algorithms for healthcare applications [83]. This could revolutionize areas like precision medicine, where highly accurate simulations and calculations are essential for developing personalized treatments. Moreover, new quantum hardware designs, such as topological qubits and improvements in quantum processors, show promise in making quantum computers more robust and capable of handling real-world medical data [84]. These advancements in hardware will push QC from experimental research into practical healthcare applications, potentially transforming fields like drug discovery, genomics, and medical diagnostics in the coming years.

**Figure 3:**
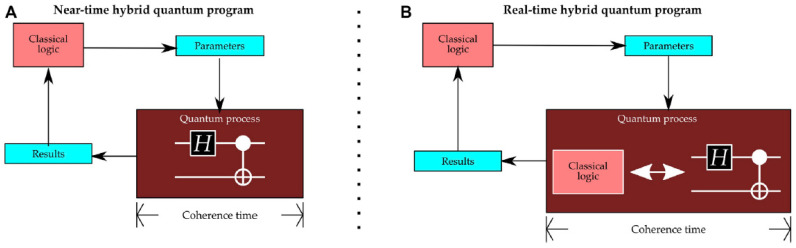
A schematic comparison between near-time hybrid programs (**A**) and the real-time hybrid quantum programs considered and implemented in this work (**B**) is shown. Notably, in the more advanced form discussed here, hybrid quantum–classical programs can make classical decisions based on the outcomes of quantum measurements. These decisions are then used to condition and control future quantum operations, all within the coherence times of quantum registers. Reproduced from reference [85] under the Creative Commons Attribution 4.0 International License (https://creativecommons.org/licenses/by/4.0/ (accessed on 2 October 2024)).

A promising direction for the near-term application of QC in healthcare is the development of quantum–classical hybrid systems. These systems leverage the strengths of both quantum and classical computing, allowing them to work together to solve complex medical problems [85]. There are two approaches to quantum programming: near-time hybrid programs and real-time hybrid quantum programs, as illustrated in Figure 3. Both methods enable faster near-time execution, although they do not achieve real-time execution of classical code while maintaining quantum state coherence.

Hybrid algorithms—such as the VQE and the quantum approximate optimization algorithm (QAOA) [86]—are designed to perform quantum computations for specific parts of a problem, while classical computers handle the rest. This approach is particularly beneficial given the current limitations in quantum hardware, as it allows for quantum advantages to be realized without requiring fully fault-tolerant quantum computers. In healthcare, hybrid systems are being explored for applications like drug discovery, genomics, and medical diagnostics, where classical computers perform data preprocessing, and quantum computers handle computationally intensive tasks, such as molecular simulations or optimization problems [87]. As quantum hardware improves, these hybrid systems will continue to evolve, allowing for greater efficiency and accuracy in healthcare algorithms. This quantum–classical collaboration is expected to be a critical bridge, enabling quantum technologies to have a more immediate impact on the healthcare sector while fully quantum systems are still under development.

The timeline for the widespread clinical adoption of QC in healthcare remains uncertain, but experts predict that its integration into mainstream medical research and clinical practice will likely unfold over the next two decades [2,31,88]. As quantum hardware and algorithms continue to improve, particularly with advancements in error correction, scalability, and quantum–classical hybrid systems, QC’s potential to solve complex healthcare problems will become more apparent. Initially, QC is expected to have the most significant impact in research environments, where it will be used for drug discovery, genomics, and molecular simulations. However, for QC to become a standard tool in clinical settings, several hurdles must be overcome, including regulatory approvals, cost reductions, and integration with existing healthcare IT systems. Experts predict that within 10 to 15 years, we will see the first quantum-assisted medical applications, such as quantum-enhanced diagnostic tools or personalized treatment optimization models, begin to appear in specialized healthcare institutions. Full-scale adoption, where QC becomes a routine part of clinical workflows, will depend on continued technological advancements and reductions in cost, potentially making QC mainstream in clinical practice by the mid-21st century.

## 7. Conclusions

QC is poised to become a transformative force in the medical field, offering unprecedented capabilities in data processing, problem-solving, and simulation. By leveraging quantum principles such as superposition, entanglement, and quantum algorithms, QC has the potential to revolutionize areas like drug discovery, genomics, medical imaging, and personalized treatment planning. These advancements will enable faster, more accurate solutions to some of the most complex problems in healthcare, where classical computing has historically struggled. As we have explored, quantum techniques such as quantum algorithms, quantum machine learning, and quantum–classical hybrid systems are already beginning to show promise in accelerating medical research and optimizing clinical applications.

The future potential of QC in healthcare is immense. From identifying new therapeutic molecules to enhancing diagnostic precision, quantum computers will allow healthcare professionals to develop more personalized and effective treatments. The ability to simulate complex biological systems at the molecular level could vastly reduce the time and cost associated with drug development, while quantum-enhanced AI models may enable earlier detection of diseases like cancer or neurodegenerative disorders. Moreover, QC’s optimization capabilities could lead to breakthroughs in creating more efficient treatment plans, especially in fields such as radiotherapy.

However, the realization of QC’s full potential in medicine hinges on continued research and development. Technological barriers, such as hardware limitations and scalability issues, still need to be addressed, and significant ethical, economic, and regulatory challenges remain. Collaborative efforts between researchers, healthcare professionals, and policymakers will be essential to ensuring that QC can be integrated safely and effectively into the healthcare system. Continued investment in quantum research will not only drive innovation but also ensure that QC becomes a key tool in solving the healthcare challenges of the future.

## Figures and Tables

**Table 1:** Comparative advantages of QC vs. classical computing in medicine. This table highlights the key areas where quantum computing outperforms classical computing, illustrating its potential to enhance data processing speed, complex problem-solving, drug discovery, genomic analysis, medical imaging, personalized medicine, AI applications, resource efficiency, and data security in the healthcare sector.

Aspect	Quantum Computing	Classical Computing
Data Processing Speed	Can process complex datasets exponentially faster due to superposition and parallelism.	Limited to sequential processing, leading to longer computation times for large datasets.
Complex Problem Solving	Efficiently solves problems involving multiple variables and probabilities, such as molecular interactions.	Struggles with NP-hard problems, requiring extensive computational resources and time.
Drug Discovery	Accelerates molecular simulations, enabling the identification of potential drug candidates more quickly.	Slower drug discovery processes, which are reliant on trial-and-error approaches and classical simulations.
Genomic Analysis	Enhances the ability to analyze complex genetic data, improving understanding of genetic interactions.	Faces limitations in handling vast genomic datasets efficiently.
Medical Imaging	Improves imaging techniques through quantum-enhanced methods, leading to higher resolution and better diagnostic capabilities.	Conventional imaging methods may not capture fine details or require extensive processing time.
Personalized Medicine	Optimizes treatment plans by considering numerous factors simultaneously, leading to tailored therapies.	Typically utilizes standard treatment protocols, which may not account for individual patient variability.
AI and Machine Learning	Enhances AI models through faster data training and improved pattern recognition in diagnostics.	Limited by classical computing power, which may slow down AI model training and analysis.
Resource Efficiency	Potentially reduces the number of computational resources needed for complex simulations and analyses.	Often requires significant computational resources and time for complex healthcare tasks.
Security and Encryption	Offers advanced encryption methods through quantum key distribution, enhancing data security.	Vulnerable to classical hacking methods, with standard encryption potentially susceptible to breaches.

**Table 2:** Summary of the current challenges and limitations of implementing quantum computing in medicine. The table highlights technological, infrastructural, ethical, and economic barriers that must be addressed for the effective integration of quantum systems into healthcare applications.

Challenges	Description	References
Limited Hardware Capabilities	Quantum computers are still in the NISQ (Noisy Intermediate-Scale Quantum) era, where qubits are highly susceptible to errors due to decoherence and noise from the environment, limiting scalability.	[72,73]
Scalability Issues	Large-scale medical simulations (e.g., drug discovery, personalized medicine, and radiotherapy) require thousands to millions of fault-tolerant qubits, far beyond current capabilities.	[74]
Specialized and Expensive Quantum Hardware	Quantum computers require highly controlled environments, such as extremely low temperatures and vacuum conditions, making them difficult and expensive to develop and maintain.	[72,80]
Integration with Clinical Settings	Quantum computers need specialized environments that are not compatible with standard healthcare infrastructure. Quantum algorithms also face challenges integrating with classical healthcare IT systems.	[75]
Workforce Training and Expertise	QC in healthcare requires a workforce skilled in both quantum mechanics and clinical applications, posing a challenge in training medical professionals.	[62]
Regulatory and Reliability Issues	Ensuring the accuracy, reliability, and regulatory approval of quantum-driven healthcare tools is a key hurdle before clinical adoption.	[62]
Data Privacy and Security Concerns	Quantum systems may break existing encryption methods, raising concerns over the security of sensitive patient data and necessitating quantum-safe encryption.	[76,77]
Ethical Issues in Quantum-enhanced AI	Quantum-enhanced AI models could introduce issues like algorithmic bias and lack of explainability, raising ethical concerns in medical decision-making.	[78,79]
High Cost of Quantum Hardware and Maintenance	The infrastructure and operational costs of quantum systems are significantly higher than classical computing, limiting adoption in resource-constrained healthcare institutions.	[13,80]
Economic Inequality	Limited availability and high costs restrict access to quantum computers, leading to economic disparities in which only well-funded organizations can benefit.	[13]

## Data Availability

No new data were created or analyzed in this study.
